# Induced pluripotent stem cell‐based assays recapture multiple properties of human astrocytes

**DOI:** 10.1111/jcmm.18214

**Published:** 2024-03-20

**Authors:** Hideki Nonaka, Takayuki Kondo, Mika Suga, Ryu Yamanaka, Yukako Sagara, Kayoko Tsukita, Naoko Mitsutomi, Kengo Homma, Ryuta Saito, Fumihiko Miyoshi, Hiromitsu Ohzeki, Masahiro Okuyama, Haruhisa Inoue

**Affiliations:** ^1^ iPSC‐based Drug Discovery and Development Team, RIKEN BioResource Research Center (BRC) Kyoto Japan; ^2^ Mitsubishi Tanabe Pharma Corporation Yokohama Japan; ^3^ Center for iPS Cell Research and Application (CiRA) Kyoto University Kyoto Japan; ^4^ Medical‐risk Avoidance based on iPS Cells Team, RIKEN Center for Advanced Intelligence Project (AIP) Kyoto Japan

**Keywords:** Alexander disease, astrocyte, autophagy, calcium, cytokine, disease complexity, induced pluripotent stem cells, migration

## Abstract

The majority of the population of glial cells in the central nervous system consists of astrocytes, and impairment of astrocytes causes various disorders. It is useful to assess the multiple astrocytic properties in order to understand their complex roles in the pathophysiology. Although we can differentiate human astrocytes from induced pluripotent stem cells (iPSCs), it remains unknown how we can analyse and reveal the multiple properties of astrocytes in complexed human disease conditions. For this purpose, we tested astrocytic differentiation protocols from feeder‐free iPSCs based on the previous method with some modifications. Then, we set up extra‐ and intracellular assessments of iPSC‐derived astrocytes by testing cytokine release, calcium influx, autophagy induction and migration. The results led us to analytic methods with conditions in which iPSC‐derived astrocytes behave as in vivo. Finally, we applied these methods for modelling an astrocyte‐related disease, Alexander disease. An analytic system using iPSC‐derived astrocytes could be used to recapture complexities in human astrocyte diseases.

## 1 INTRODUCTION

Astrocytes are major glial cells in the central nervous system (CNS), and their aberrations are found in many human diseases including strokes, neuroinflammatory diseases, neurodegenerative diseases and developmental diseases. Accumulated evidence has shown that, in a disease condition, astrocytes play multiple roles and form complexities of human diseases, since they are known to have various functions affecting the brain environment, such as cytokine secretion, neural maturation and maintenance of cellular homeostasis.^1^ Therefore, for understanding the complexity of human diseases, assessing various astrocytic properties is crucial.

After the advent of induced pluripotent stem cells (iPSCs), besides rodent models, astrocytes derived from patient iPSCs have been a research resource for human astrocyte diseases. Protocols of astrocyte differentiation from human iPSCs have been reported.^2^, ^3^, ^4^, ^5^, ^6^, ^7^, ^8^ Although we previously reported a differentiation method for astrocytes as well as a disease modelling capability protocol,^9^ methods for the induction and analysis of multiple properties of human astrocytes have yet to be established. In the present study, we optimized the astrocytic differentiation protocol reported previously for feeder‐free iPSC cultures.^9^ Then, we set up the analytic methods to estimate astrocytic properties including cytokine release, calcium influx, autophagy induction and migration in a dish. These assay systems recaptured iPSC‐derived multi‐functions of astrocytes.

Finally, by using an astrocyte‐related disease, Alexander disease (AxD) patient‐derived iPSCs, we detected disease phenotypes, including glial fibrillary acidic protein (GFAP)‐aggregates, and gene expression changes. AxD was studied in the field of stem‐cell research. This was because the responsible gene of the disease, GFAP, is a representative marker of mature, reactive astrocytes, and the disease shows a histopathological feature named Rosenthal fibre, an aggregate mainly consisting of GFAP.^10^, ^11^ These disease characteristics matched the research validated whether iPSC‐derived astrocytes show the pathological features or not, and some studies using iPSC‐derived astrocytes have been reported.^12^, ^13^, ^14^ In the current study, we showed that the presented methods for induction and analysis of human astrocytes can be applicable to reveal the pathophysiology of the astrocyte disease.

## 2 MATERIALS AND METHODS

### 2.1 Differentiation of human iPSCs into astrocytes

Established human iPSCs (HPS1046 and HPS3529) were provided by RIKEN‐BRC (https://web.brc.riken.jp/en/) through the National BioResource Project of MEXT/AMED, Japan (Tables S1). The present study was approved by the Ethics Committee of BioResource Research Center, RIKEN (approval no. = Tsukuba2019‐3, Tsukuba29‐1). They were maintained in StemFit AK02N (Ajinomoto, Tokyo, Japan) on iMatrix‐511 (Matrixome, Osaka, Japan)‐coated plates. We differentiated iPSCs into astrocytes according to the method previously reported protocol,^9^ with some modifications, as below. iPSCs were dissociated by Accutase (Nacalai Tesque, Kyoto, Japan) with 10 μM Y‐27632 (Nacalai Tesque) and quickly reaggregated in U‐bottom 96‐well plates (Thermo Fisher Scientific, Waltham, MA) by centrifugation to form embryonic bodies (EBs). These EBs were cultured in ‘DFK5DSY medium’ (DFK5DSY; DMEM/F12 GlutaMAX supplement (Thermo Fisher Scientific) with 5% v/v KnockOut Serum Replacement (KSR) (Thermo Fisher Scientific), 2 μM dorsomorphin (Sigma‐Aldrich, St. Louis, MO), 10 μM SB431542 (Cayman Chemical, Ann Arbor, MI), 10 μM Y‐27632, Non‐Essential Amino Acids Solution (NEAA) (Thermo Fisher Scientific), 0.1 mM 2‐mercaptoethanol (Thermo Fisher Scientific) and penicillin–streptomycin (Thermo Fisher Scientific)) at day 0–8. After that, those EBs were plated on Matrigel‐coated six‐well culture plates and cultured in ‘DFN2D medium’ (DFN2D; DMEM/F12 GlutaMAX supplement with N2 supplement (Thermo Fisher Scientific), 2 μM dorsomorphin, NEAA, 0.1 mM 2‐mercaptoethanol and penicillin–streptomycin) at day 8–14 and cultured in ‘DFN2D medium’ with 10%KSR at days 14–22. The cells at day 22 were Nestin‐positive and highly expressed Sox2 and Pax6 mRNA. The day 22 cells were dissociated by Accutase with 10 μM Y‐27632, replaced on matrigel (Corning, Tewksbury, MA)‐coated 12‐well culture plates and cultured in ‘Neurobasal FULL medium’ (Neurobasal medium (Thermo Fisher Scientific) with 0.5× N2 supplement, 0.5× B27 without VA (Thermo Fisher Scientific), 10 ng/mL BDNF (Peprotech, Rocky Hill, NJ), 10 ng/mL GDNF (Peprotech), 10 ng/mL NT3 (Peprotech), GlutMAX (Thermo Fisher Scientific) and penicillin–streptomycin) at days 22–90. At day 90, cells were dissociated with Accutase with 10 μM Y‐27632 and replaced on Matrigel‐coated 12‐well culture plates and cultured in ‘Astrocyte medium’ (DMEM/F12 GlutaMAX supplement with N2 supplement, 10 ng/mL EGF (Peprotech), 10 ng/mL basic FGF (Peprotech), 2 μg/mL heparin (Nacalai Tesque) and penicillin–streptomycin) at days 90–200. Cells reaching confluence were passaged and replated on 12‐well plates at 5, 15, 30 × 10^4^ cells per well each time. At the passage, the cells were seeded with three different cell densities, and we continued the culture using the lowest seeding density wells to the extent that no obvious cell death occurred. The choice of low‐density seeding conditions accelerated the negative selection of neurons with weak adhesion to the plate, which could not adhere to the top of the astrocytes. Astrocytes from human brain were purchased from ScienCell Research Laboratories (Carlsbad, CA) and cultured in Astrocyte Medium (ScienCell) on plates coated with poly‐l‐Lysine (Sigma‐Aldrich).

### 2.2 Immunocytochemistry

Cells were fixed with 4% paraformaldehyde, permeabilized with 0.1% triton X‐100 and blocked by 1% normal goat serum, 1% BSA and 0.1% tween20 in PBS for 1 h. Those samples were then incubated at 4°C overnight with primary antibodies diluted in PBS containing 1% BSA and 0.1% tween20. Next, those samples were incubated with secondary antibodies at room temperature for 1 h. Hoechst 33342 (Doujin Chemical, Kumamoto, Japan) was used to counterstain the nuclei. The following primary and secondary antibodies were used: anti‐S100β (Abcam, Cambridge, UK, ab52642, 1:200), GFAP (Cell Signalling Technology, Beverly, MA, 3670, 1:1000), NESTIN (Millipore‐Sigma, St. Louis, MO, MAB5326, 1:500), Alexa Fluor 488‐conjugated Goat anti‐Rabbit IgG (H + L) (Thermo Fisher Scientific, A‐11034, 1:1000) and Alexa Fluor 555‐conjugated Goat anti‐Mouse IgG (H + L) (Thermo Fisher Scientific, A‐21422, 1:1000). After immunostaining steps, cells were analysed by Opera Phenix (PerkinElmer, Waltham, MA) with 10× or 40× objective lenses, followed by quantification for the positivity of astrocytic markers using Harmony software (PerkinElmer). When calculating GFAP aggregates, the images were taken using a 40× objective lens and the round areas showing strong intensity against the backgrounds were counted (Common Threshold: 0.90, Area > 10 pixels, Roundness > 0.80).

### 2.3 Electrochemiluminescence assays for cytokines

Cells were plated at 5 × 10^4^ cells per well in 96‐well plates coated with Matrigel. To control the number of cells, the assay was performed relatively soon after seeding. Therefore, the seeding density was higher than that in other assays or usual passages. Three days after plating, culture media were replaced with 100 μL/well of Astrocyte Media containing IL‐1β (Peprotech) or TNFα (Peprotech), harvested 24 h after replacement. Twenty‐five microlitres of conditioned media was subjected to electrochemiluminescence (ECL)‐ELISA analysis by using a V‐PLEX Custom Human Biomarkers kit (IL‐1β, IL‐6, TNF‐α, IL‐4 and GM‐CSF) (Meso Scale Discovery, Rockville, MD) and MESO SECTOR S600 (Meso Scale Discovery). Cytokine concentrations were calculated based on the kit's standard calibration curve using a four‐parameter logistic fit by applying MSD Discovery workbench 4.0 software (Meso Scale Discovery).

### 2.4 Calcium measurements

Cells were plated at 5 × 10^4^ cells per well in 96‐well plates coated with Matrigel. To control the number of cells, the assay was performed relatively soon after seeding. Therefore, the seeding density was higher than that in other assays or usual passages. Two days after plating, cells were loaded with 2 μM Fluo‐8 AM (AAT Bioquest, Sunnyvale, CA) and incubated at 37°C for 45 min. After washing with PBS, HBSS/HEPES with 1 mM probenecid was added (Thermo Fisher Scientific). ATP (Nacalai Tesque)‐inducing calcium influx was measured by FDSS/μCELL (Hamamatsu, Hamamatsu, Japan) for 5 min (0.5 s intervals) at 37°C. The excitation wavelength was 470 nm and the emission wavelength was 540 nm. When adding ATP, 20 μL of a fivefold concentrated ATP solution was added to 80 μL of the culture supernatant, and the fluorescence intensity was continuously measured.

### 2.5 Scratch‐wound assay

Cells were cultured in IncuCyte Imagelock 96‐well plates (Sartorius, Göttingen, Germany) coated with Matrigel until confluent. Then, cells were scratched by 200 μL pipette tip and a straight line was made. Cells were monitored by IncuCyte S3 (Sartorius) every 3 h and confluency map was calculated by using IncuCyte software (IncuCyte version 2021C, Sartorius).

### 2.6 Autophagy detection

We used 1 μM DALGreen reagent (Dojin Chemical) according to the manufacturer's protocol. Starvation was induced by DMEM/F‐12 without any supplements and growth factors. The cells were monitored by IncuCyte S3 and the total integrated intensity/cell area was calculated.

### 2.7 Genotyping

Genomic DNA of iPSCs was extracted by PureLink Genomic DNA Mini Kit (Thermo Fisher Scientific) and amplified by PCR using PrimeSTAR GXL DNA Polymerase (TaKaRa Bio, Shiga, Japan) and Veriti thermal cycler (Thermo Fisher Scientific). The thermal conditions for PCR were 30 cycles of 10 s at 98°C, 15 s at 60°C and 1 min at 68°C. The amplified PCR products were extracted from agarose gel by PureLink Quick Gel Extraction Kit (Thermo Fisher Scientific). Sequencing analysis was done by 3500 xL Genetic analyser (Thermo Fisher Scientific). Forward (F) and reverse (R) PCR primers were as follows: F, 5′‐tgaccaggtgttgtgctagg‐3′, R, 5′‐ccgacttggggaggtttcg‐3′.

### 2.8 Quantitative RT‐PCR

cDNA samples for quantitative RT‐PCR were prepared by SuperPrep Cell Lysis Kit instructions attached to the kit (Toyobo, Osaka, Japan). cDNA was used as template for quantitative PCR analysis, using SYBR Green II (TAKARA, Kusatsu, Japan) and StepONE plus (Thermo Fisher Scientific). To perform relative quantification, the comparative threshold (*C*_t_) cycle method was used. GAPDH was used as housekeeping control gene to normalize target genes. The fold change in gene expression profile was referred to iPSCs sample. Primer sequences (5′–3′) for amplification of qPCR were the following: NANOG‐Fw GATTTGTGGGCCTGAAGAAA, NANOG‐Rv CTTTGGGACTGGTGGAAGAA; PAX6‐Fw AGTTCTTCGCAACCTGGCTA, Pax6‐Rv ATTCTCTCCCCCTCCTTCCT; NESTIN‐Fw TCCAGGAACGGAAAATCAAG, NESTIN‐RV GCCTCCTCATCCCCTACTTC; MAP2‐Fw CAGCAGGTGGGGAATCAG, MAP2‐Rv GCAGGAATCTTTGACAAGGTAGA; S100B‐Fw GGAAGGGGTGAGACAAGGA, S100B‐Rv GGTGGAAAACGTCGATGAG; GFAP‐Fw GTCTGTGTCAGAAGGCCACC, GFAP‐Rv GTGCTCCTGCTTGGACTCC.

### 2.9 Protein measures

To assess protein levels in iPSC‐derived astrocytes, a ‘Wes’ Simple Western system (ProteinSimple, Inc., San Jose, CA), a capillary‐based semiquantitative protein analysis tool, was used. All iPSC‐derived astrocytes were lysed with RIPA buffer (Wako Pure Chemicals, Osaka, Japan). Samples were centrifuged, and the supernatant was collected. All extracted protein samples were diluted to the same protein concentration of 0.5 μg/mL and were subjected to 12–230 kDa Separation Module (ProteinSimple, Inc.) with specific primary antibodies to detect LC3 (Novus Biologicals, LLC, Littleton, NB600‐1384, CO), p62/SQSTM1 (MBL, PM045, Woburn, MA), S100B, GFAP (Novus Biologicals, LLC, Littleton, NB300‐141) and β‐actin (Novus Biologicals, LLC, Littleton, NB110‐67828). Detected bands were analysed by using COMPASS 6.3.0 software (ProteinSimple, Inc.).

### 2.10 Statistical analysis

All data were shown as mean ± SEM. For comparisons of the mean between two groups, statistical analysis was performed by unpaired *t*‐test. For comparisons of the mean among more than three groups, statistical analysis was performed by one‐way ANOVA followed by Tukey's multiple comparison test. All analyses were conducted by GraphPad Prism 101.2 (GraphPad Software, San Diego, CA). *p* Values <0.05 were considered statistically significant.

## 3 RESULTS

### 3.1 Differentiation of iPSCs into astrocytes

**FIGURE 1:**
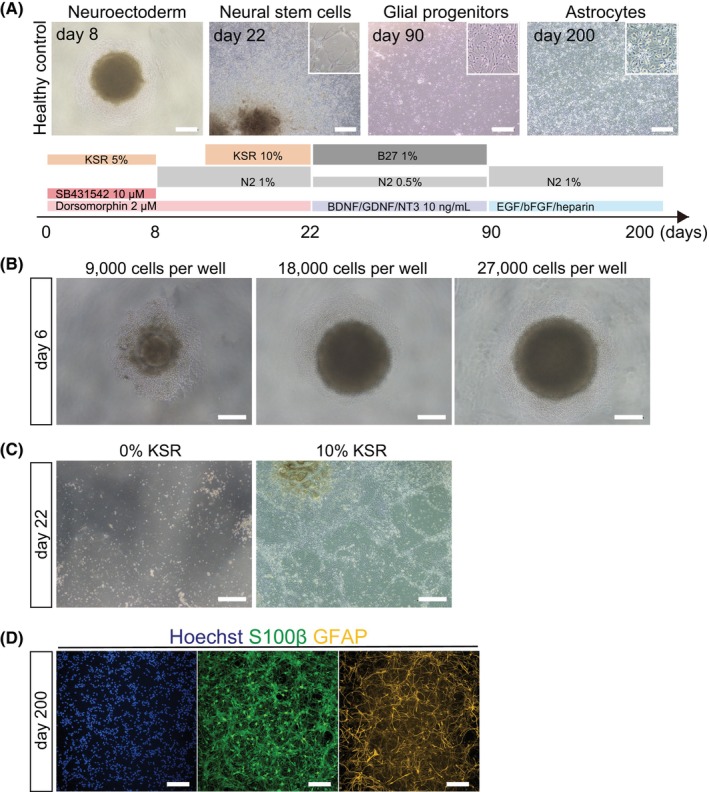
iPSCs differentiated into astrocytes. (A) Schematic procedures for astrocytic differentiation. Scale bar = 200 μm (day 8) or 500 μm (others). (B) Morphology of embryoid body (EB) at each cell number. Scale bar = 200 μm. (C) Effect of 10% KSR against cell detachment. Scale bar = 500 μm. (D) Immunostaining images of astrocytic marker protein S100β and GFAP. Scale bar = 200 μm. Data represent mean ± SEM (*n* = 3, from different experimental batches).

We differentiated feeder‐free iPSCs to astrocytes by modifying the previously reported method of on‐feeder iPSC culture (Figure 1A). Briefly, first we differentiated iPSCs (Figure S1A,B) to neural stem cells via EBs. There were some key points during the optimization. We examined multiple conditions regarding the number of iPSCs seeded to form EBs on day 0, the time point at which differentiation begins. When iPSCs were seeded with 9000 or 18,000 cells per EB, about half of the wells failed to form EBs, some EBs collapsed and the size of the EBs was variable (Figure 1B). However, under the seeded cell number condition of 27,000 cells per EB, the size of EBs tended to be larger and EBs formed well with good reproducibility (Figure 1B). Around day 14, a critical problem of cells becoming detached from plates occurred. We could prevent this by adding 10% KSR to the medium (Figure 1C). On day 22, a large number of cells were migrating from EBs attached to the bottom of the plate, and these cells were positively stained for NESTIN, a marker for neural stem cells (Figure S1C). By these modifications, we could differentiate iPSCs cultured in feeder‐free systems into astrocytes (Figure 1D).

### 3.2 Astrocytic multifunction of iPSC‐derived astrocytes

Assay systems such as cytokine release^15^, ^16^ and calcium influx^1^ were used to evaluate astrocytic functions and reveal the pathology of astrocyte‐related diseases.

First, we tested the cytokine releasing capacity of iPSC‐derived astrocytes.^17^, ^18^ It is known that inflammatory stimuli such as IL‐1β or TNF‐α induces cytokine secretions in primary and iPSC‐derived astrocytes. Therefore, we treated iPSC‐derived astrocytes with IL‐1β or TNF‐α and measured the factors (IL‐1β, IL‐6, TNF‐α, IL‐4 and GM‐CSF) in the medium. Cleary, all of them were increased by each stimulus (Table 1). This suggested that our iPSC‐derived astrocytes had the ability to secrete various factors and that they could be used to study inflammatory diseases related to these cytokines.

**TABLE 1:** iPSC‐derived astrocytes released IL‐1β, IL‐6, TNF‐α, IL‐4 and GM‐CSF by treatment of IL‐1β or TNF‐α.

	Released cytokines					
		IL‐1β (pg/mL)	IL‐6 (pg/mL)	TNF‐α (pg/mL)	IL‐4 (pg/mL)	GM‐CSF (pg/mL)
Cell treatment	Control medium	0.006 ± 0.001	0 ± 0	0.04 ± 0.02	0.005 ± 0.001	0.1 ± 0
	IL‐1β 100 ng/mL		468 ± 34***	2.32 ± 0.06***	0.301 ± 0.033***	70.2 ± 4.9***
	TNF‐α 100 ng/mL	0.101 ± 0.008***	4 ± 0***		0.121 ± 0.006***	6.3 ± 0.3***

*Note*: Data represent mean ± SEM (*n* = 3).

*** *p* < 0.001 compared with control medium.

**FIGURE 2:**
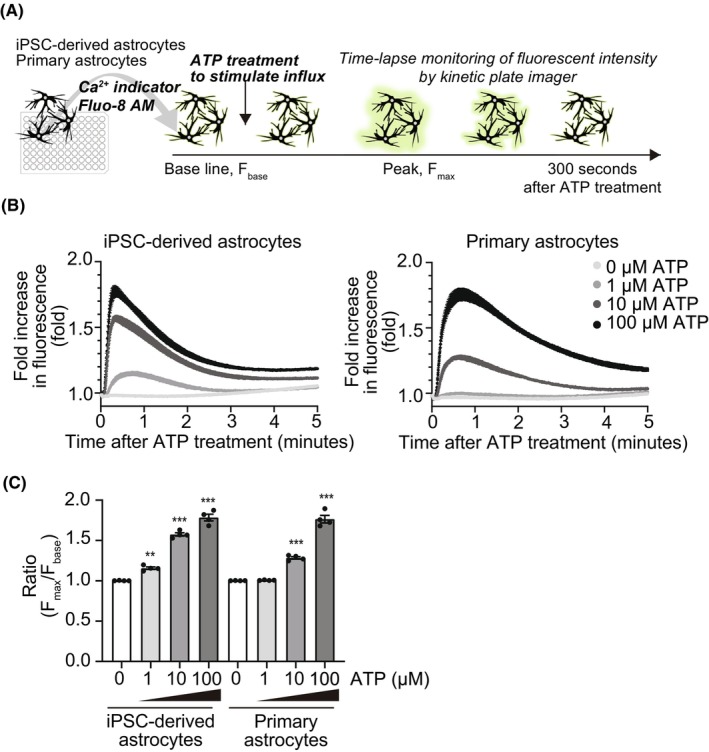
Calcium measurement after ATP treatment assessed the intracellular calcium regulatory function of iPSC‐derived astrocytes. (A) Schematic procedures of calcium assay. (B, C) Calcium response induced by ATP treatment. Kinetics (B) and peak ratio (C) of iPSC‐derived and primary astrocytes' fold increase in fluorescence. **p < 0.01, ***p < 0.001 compared with 0 μM ATP treatment. Data represent mean ± SEM (*n* = 4, from different experimental batches).

Next, we tested the ATP‐inducing calcium influx. This function was previously evaluated in iPSC‐derived astrocytes as one of the representative astrocytic assays.^5^, ^19^ ATP activates P2X and P2Y receptors and the cellular calcium level increases. Our iPSC‐derived astrocytes could respond to ATP treatment and increase the cellular calcium level. It rose to the top level in about 0.5–1 min and decreased to near bottom level about 5 min after treatment (Figure 2B). We also tested primary astrocytes and similar responses were observed (Figure 2C).

**FIGURE 3:**
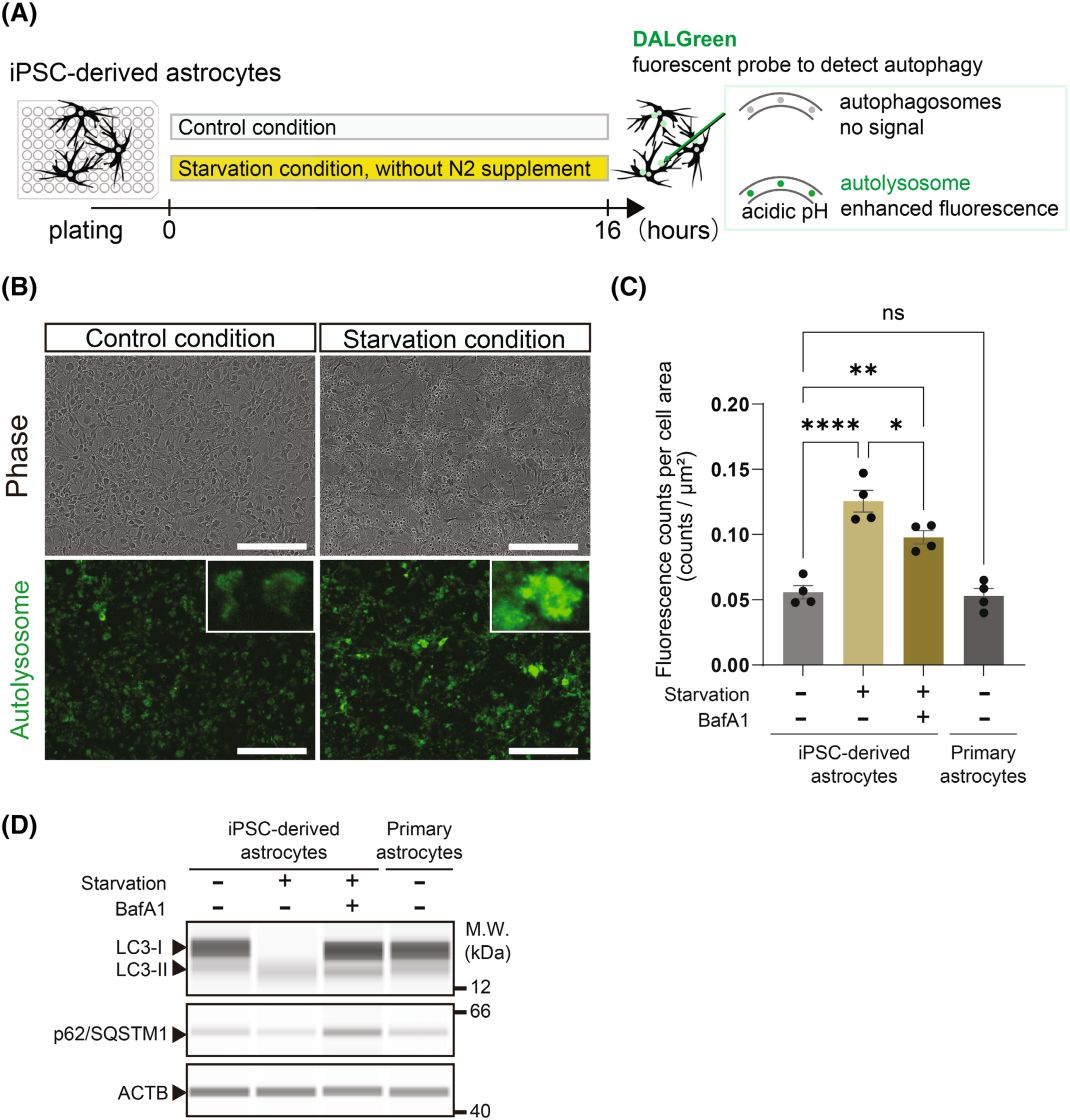
Autolysosome imaging after growth factor deprivation captured the autophagy induction in iPSC‐derived astrocytes. (A) Schematic procedures of autophagy induction assay. (B) Representative images of cells and autolysosomes on day 2 with or without growth factor deprivation. (C) Fluorescence counts per well of 96‐well plate at 16 h after initiation of assay. Astrocytes were treated with control, starvation and starvation with 10 nM bafilomycin A1 (BafA1) condition. The data were analysed by one‐way ANOVA (*F* [3, 12] = 21.19, *p* < 0.0001), followed by Tukey's multiple comparisons test. *p < 0.05, **p < 0.01, ****p < 0.001, ns, not significant. Data represent mean ± SD (*n* = 4, from different experimental replicates). (D) Astrocytes were treated with control, starvation and starvation with 10 nM bafilomycin A1 (BafA1) condition. Cells were lysed and subjected to immunoblot analysis at 16 h after initiation of assay. M.W., molecular‐weight size marker.

In some neurodegenerative diseases, autophagy induction and autolysosome clearance were impaired,^20^ and in regard to not only neurons but also astrocytes, abnormal autophagy was reported.^21^, ^22^ To induce autophagy experimentally, growth factor deprivation and endoplasmic reticulum stress loading are used. Then, we cultured our iPSC‐derived astrocytes in medium without growth factor and supplement to induce autophagy (Figure 3A). In the medium lacking nutrition, astrocytes changed their morphology and the formation of autolysosomes was detected (Figure 3B). When quantifying the DALGreen assay results, we added bafilomycin A1 (BafA1), an autophagy inhibitor, to confirm whether autophagy occurs under the starvation conditions detected by DALGreen. We also compared iPSC‐derived astrocytes with human primary astrocytes as a negative control. Autolysosome formation, detected by increased fluorescence intensity of DALGreen, was increased under starvation conditions (Figure 3C). Consistent with previous reports, the addition of BafA1 inhibited lysosome degradation^23^ by preventing autolysosome maturation and reduced the fluorescent signal.^24^, ^25^ Additionally, iPSC‐derived astrocytes and human primary astrocytes had similar DALGreen fluorescence intensity. Next, we examined the protein levels of autophagy‐related molecules to examine whether autophagy was activated under these experimental conditions. Starvation conditions, which enhance the conversion of microtubule‐associated proteins 1A/1B light chain 3B (LC3) from LC3‐I to LC3‐II, resulted in an increase in LC3‐II (Figure 3D) as previously reported.^23^, ^26^ The addition of BafA1 increased the protein level of LC3‐II, indicating that BafA1 inhibited the degradation of lysosomes. Similarly, we also examined the protein levels of ubiquitin‐binding protein p62 (p62), also known as sequestosome‐1 (SQSTM1), which is used to evaluate the autophagy status because p62 binds directly to LC3 and is selectively degraded by autophagy.^23^ Starvation and BafA1 also increased p62 protein levels. Furthermore, there were no significant differences in LC3 and p62 protein levels between iPSC‐derived astrocytes and human primary astrocytes (Figure 3D). These results showed that changes in DALGreen fluorescence intensity reflected the molecular process of autophagy.

**FIGURE 4:**
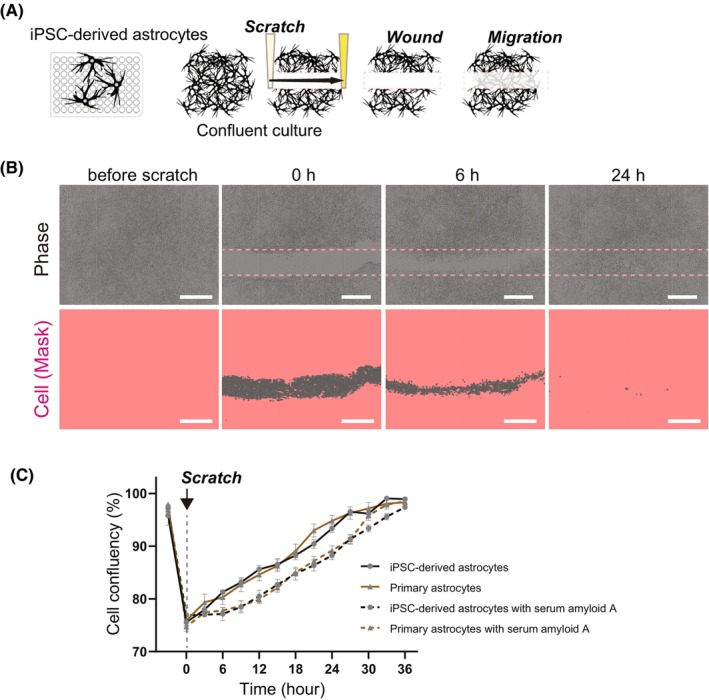
Time imaging after scratch quantified the migratory function of iPSC‐derived astrocytes. (A) Schematic procedures of scratch assay. (B) Representative images of cells (upper) and cell yellow masks (lower) at each time point after scratching. Dotted lines show the site scratched. Scale bar = 500 μm. (C) Change of cell confluency after scratching over time. Immediately after scratching, cell confluency of iPSC‐derived astrocytes (black line) and human primary astrocytes (brown line) was recorded every 3 h. Additionally, conditions with 10 μg/mL serum amyloid A (SAA) (dashed line) were recorded in the same way. Data represent mean ± SD (*n* = 4, from different experimental replicates).

And finally, astrocytes are known to extend their processes and migrate to the wound region after traumatic injury, and this process affects the recovery.^27^ In vitro scratch assay is used as a method for assessing cell migration^28^ and was reported to be applicable for primary astrocytes.^29^ Therefore, we tried to investigate whether the scratch assay is applicable to iPSC‐derived astrocytes or not. After becoming confluent, we created a straight‐line scratch (Figure 4A). Our iPSC‐derived astrocytes responded to the scratching injury and migrated to the lesion site, filling the scar (Figure 4B,C). We compared the migration status of iPSC‐derived astrocytes to verify whether they have mature functions similar to human primary astrocytes. Furthermore, by adding serum amyloid A (SAA), which has been reported to inhibit migration,^30^ we simultaneously evaluated responsiveness to environmental conditions. When comparing iPSC‐derived astrocytes and primary astrocytes, no difference was seen in their migration function (Figure 4C). It was also revealed that the migration of both astrocytes was inhibited by the addition of SAA for several hours after the start of the assay (Figure 4C). These results revealed that iPSC‐derived astrocytes have an important migration function so as to perform a role in the brain, and that they are also suitable for this functional evaluation.

This result suggested, for example, that iPSC‐derived astrocytes could be used to study the disease‐changing condition in case of traumatic injury.

### 3.3 Alexander disease modelling by using iPSC‐derived astrocytes

**FIGURE 5:**
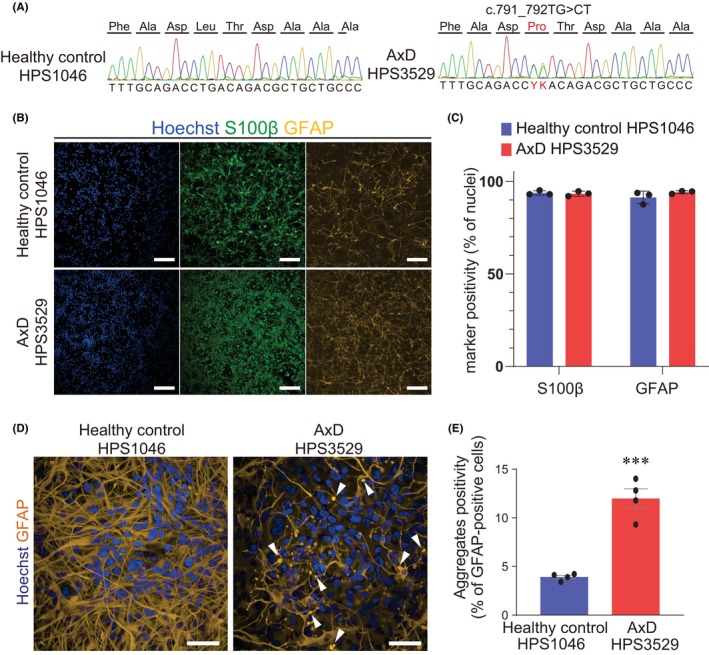
Alexander disease patient's iPSC‐derived astrocytes showed disease‐like phenotype. (A) DNA sequencing of genomic DNA of healthy control (HPS1046) and Alexander disease (AxD) (HPS3529) iPSCs. (B) Immunostaining images of astrocytic marker protein S100β and GFAP in both iPSC‐derived astrocytes of a healthy control clone and an AxD clone. Scale bar = 200 μm. (C) S100β or GFAP positivity against total Hoechst (%) was quantified. Data represent mean ± SEM (*n* = 3). (D) Representative images of GFAP structure. In healthy control, GFAP showed filamentous structures, but dot‐like arrowheads in AxD. Scale bar = 50 μm. (E) GFAP aggregates positivity. ***p < 0.001 compared with healthy control. Data represent mean ± SEM (*n* = 4, from different experimental batches).

We showed iPSC‐derived astrocytes with multiple properties. iPSC‐derived astrocytes can be used for assay systems to study human astrocytes and astrocyte‐related diseases. Finally, we estimated the capability of our iPSC‐derived astrocytes for disease modelling by using AxD patients' iPSCs (Figure S2A,B). In a previous study with an iPSCs feeder system,^9^ AxD disease patients' iPSC‐derived astrocytes showed the pathological feature of Rosenthal fibre‐like GFAP aggregates. Therefore, in this study, we used the one representative AxD patient iPSCs carrying the mutation of GFAP (c.791_792TG>CT) (Figure 5A)and differentiated them into astrocytes. On day 22, a large number of cells were migrating from EBs attached to the bottom of the plate, and these cells were NESTIN‐positive, a marker for neural stem cells (Figure S2C). To assess the variation in differentiation processes among different iPSC clones, we characterized the gene expression patterns at each stage of differentiation from iPSCs derived from healthy controls and AxD patients into astrocytes. When iPSCs were transformed into neural stem cells, the expression of NANOG, an undifferentiated marker, decreased and the expressions of PAX6 and NESTIN, markers of neural stem cells, increased (Figure S3A). Similar to the neural stem cell marker, the expression of neuronal marker MAP2 was also elevated on day 22, but decreased on day 117, when the number of neurons gradually decreased and differentiated into astrocytes (Figure S3A). By the final stage, day 200, neural stem cells and neuronal markers were significantly reduced. The expression of astrocyte markers S100B and GFAP had increased markedly by day 200 (Figure S3A). At each stage of differentiation, there were differences in the expressions of neural stem cells or neuronal markers between healthy control and AxD iPSCs, reflecting clonal variations of iPSCs. However, S100B and GFAP expressions were similar on day 200, when astrocytes were purified. Protein levels of S100B and GFAP were also similar between the different iPSC‐derived astrocytes (Figure S3B).

These astrocytes highly expressed the astrocytic markers S100 β (93.7 ± 0.7% of cells for healthy control, 93.4 ± 0.8% of cells for AxD) and GFAP (91.3 ± 1.9% of cells for healthy control, 94.2 ± 0.5% of cells for AxD) (Figure 5B,C) and showed GFAP‐aggregate‐like structures (Figure 5D,E). These structures were similar to those in our previous study and other published reports.^5^, ^9^, ^12^, ^13^ Established assays were also compatible with AxD iPSCs and assessed the autophagy and migratory function of AxD astrocytes (Figure S4A,B). These results showed that analysis of human astrocytes can be applied to construct disease models of astrocytic disorders and, down the road, elucidate their pathophysiologic features.

## 4 DISCUSSION

We established the induction and analytic system of multiple properties of human astrocytes using feeder‐free iPSCs. We have proposed methods for comprehensively evaluating the functions of iPSC‐derived astrocytes. These assessment systems allow us to understand how astrocytes function in the CNS and how they are impaired to reveal disease‐associated properties in the patient brain.

Since astrocytes are the major population of glial cells in the CNS, astrocyte responses have a great impact on CNS integrity. Astrocytes have been reported to control inflammation after receiving signals from other types of immune‐related cells, such as microglia.^16^ In the current study, we set up the induction and analytic system of extra‐ and intracellular properties of iPSC‐derived astrocytes. To monitor the extracellular secretion of cytokines, we quantified the concentration of IL‐1β or IL‐6, TNF‐α, IL‐4 and GM‐CSF released in the culture media and evaluated the characters when triggering inflammatory responses. These secreted factors affect various cells and tissues including, besides microglia, neurons and the blood–brain barrier, T/B cells and astrocytes enlarge the neuroinflammatory process. In this way, cytokines released from astrocytes would control inflammation in the brain by stimulating or suppressing inflammation, in collaboration with other immune‐related cells.^31^, ^32^

To simulate communication signals from neurons or vascular endothelial cells by a neurotransmitter, we assessed the ATP‐induced calcium response of iPSC‐derived astrocytes. In general, iPSC‐derived cells are often less functional than the primary cells because they are reprogrammed. However, in this study, iPSC‐derived astrocytes showed slightly higher reactivity to ATP than primary astrocytes. This might be due to differences in the developmental stage or characteristics of gene expression, as previously reported.^18^ Some studies using in vivo models showed that disturbance of calcium homeostasis was related to diseases such as ischemia, neurodegenerative diseases and psychiatric disorder.^22^, ^33^ For instance, astrocytes of AD model mice increased the ATP‐induced calcium response^34^ and the blockade of purinoreceptor rescued gliosis.^35^ Astrocytes of AxD model mice showed that abnormal calcium signalling was linked to the gliosis,^36^ and iPSCs having a mutation of AxD‐derived astrocytes showed abnormal ATP‐induced calcium response.^5^ We also assayed induced autophagy in human iPSC‐derived astrocytes by deprivation of growth factors in order to simulate protein homeostasis in astrocytes. Autophagy in astrocytes, not only in neurons, was also involved in the clearance of abnormal protein related to diseases such as Alzheimer's disease and Parkinson disease. Mutations of genes related to autophagy were found as causable genes of some diseases such as Parkinson disease and multiple sulfatase deficiency. Therefore, iPSC‐derived astrocytes could also be used to reveal these autophagy‐related diseases.

Gliosis at the time of brain injury is the result of astrocytes migrating from the peri‐lesion area and enveloping the lesion in order to minimize it. Scratch assay had been used to evaluate the migration ability of endothelial cells, tumour cells, neural precursors and primary astrocytes. In the human body and in in vivo models, GFAP‐positive astrocytes surround the lesion sites after traumatic injury, and mice deficient of both GFAP and vimentin damages the glial scar formation capacity post‐injury.^37^ These various reports showed the involvement of astrocytes in the reaction to and the recovery from brain injury. Therefore, study projects using iPSC‐derived astrocytes seem to be useful for revealing at least a part of the response after injury, or for research concerning diseases exacerbated by injury.

In recent years, the importance of astrocyte function and pathogenesis has become clear. There is also growing interest in the development of therapies that target astrocytes. All the experimental systems that we propose to evaluate properties of astrocytes can be adapted for high‐throughput screening. In the future, it is expected that the development of therapeutic methods will proceed together with an index of astrocytic functions.

In the current study, we tested the disease modelling capability of our iPSC‐derived astrocytes by using AxD patients. Although AxD was mainly caused by a mutation of GFAP, clinical symptoms of AxD are heterogeneous, and multiple properties of astrocytes may contribute to the diverse disease conditions.

In conclusion, the presented methods for induction and analysis of human astrocytes can be applied to reveal the pathophysiology of astrocyte disease. It is crucial to understand the multiple properties of human astrocytes that contribute to the disease complexity in the CNS.

## AUTHOR CONTRIBUTIONS

**Hideki Nonaka:** Investigation (equal); methodology (equal); writing – original draft (equal). **Takayuki Kondo:** Data curation (equal); investigation (equal); methodology (equal); visualization (equal); writing – original draft (equal); writing – review and editing (equal). **Mika Suga:** Methodology (supporting); resources (supporting); supervision (equal); validation (equal). **Ryu Yamanaka:** Conceptualization (supporting); supervision (equal); writing – review and editing (supporting). **Yukako Sagara:** Methodology (supporting); validation (supporting); visualization (supporting). **Kayoko Tsukita:** Methodology (supporting); visualization (supporting); writing – review and editing (supporting). **Naoko Mitsutomi:** Supervision (supporting); validation (supporting); writing – review and editing (supporting). **Kengo Homma:** Supervision (supporting); validation (supporting); writing – review and editing (supporting). **Ryuta Saito:** Supervision (supporting); writing – review and editing (supporting). **Fumihiko Miyoshi:** Supervision (supporting); writing – review and editing (supporting). **Hiromitsu Ohzeki:** Conceptualization (equal); supervision (equal); validation (equal). **Masahiro Okuyama:** Conceptualization (equal); data curation (equal); supervision (equal); writing – review and editing (supporting). **Haruhisa Inoue:** Conceptualization (lead); data curation (supporting); funding acquisition (lead); investigation (lead); writing – review and editing (equal).

## CONFLICT OF INTEREST STATEMENT

This study was a joint research programme with Mitsubishi Tanabe Pharma Corporation, and so was in part funded by it. Hideki Nonaka, Ryu Yamanaka, Naoko Mitsutomi, Kengo Homma, Ryuta Saito, Fumihiko Miyoshi, Hiromitsu Ohzeki, and Masahiro Okuyama are employees of Mitsubishi Tanabe Pharma Corporation. Takayuki Kondo, Mika Suga, Yukako Sagara, Kayoko Tsukita and Haruhisa Inoue have no conflicts of interest to declare that are relevant to the content of this study.

## Supporting information


Figure S1–S5

Table S1

## Data Availability

The data that support the findings of this study are available from the corresponding author upon reasonable request.
