# Supplementary figures and images for: Induced pluripotent stem cell‐based assays recapture multiple properties of human astrocytes
Source: J Cell Mol Med. 2024 Mar 20;28(7):e18214. doi: 10.1111/jcmm.18214 (PMC10955154; doi:10.1111/jcmm.18214)

# Figure S1

---

(A)

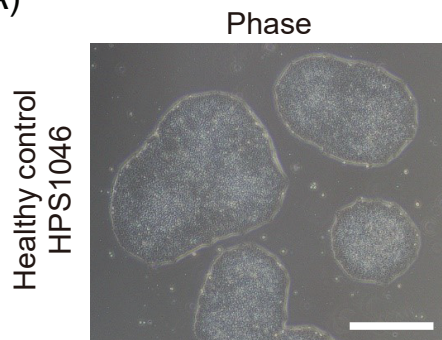

(B)

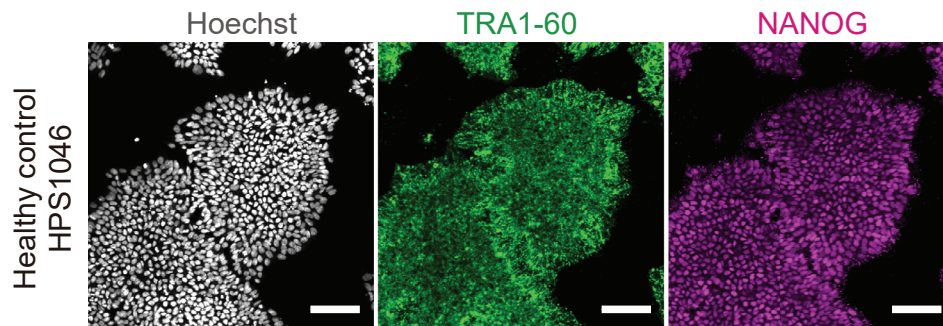

(C)

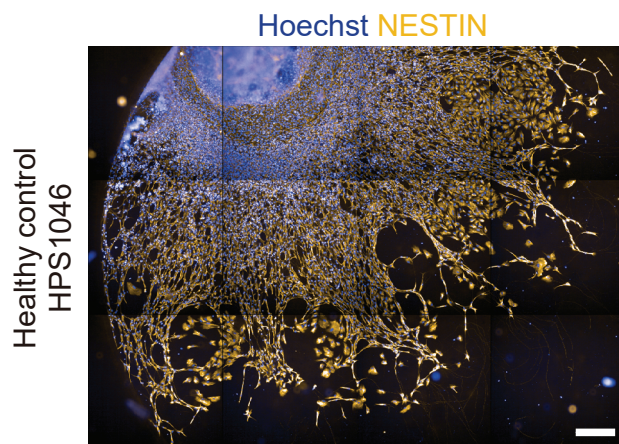

# Figure S2

---

(A)

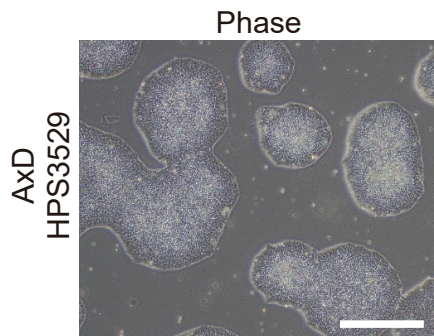

(B)

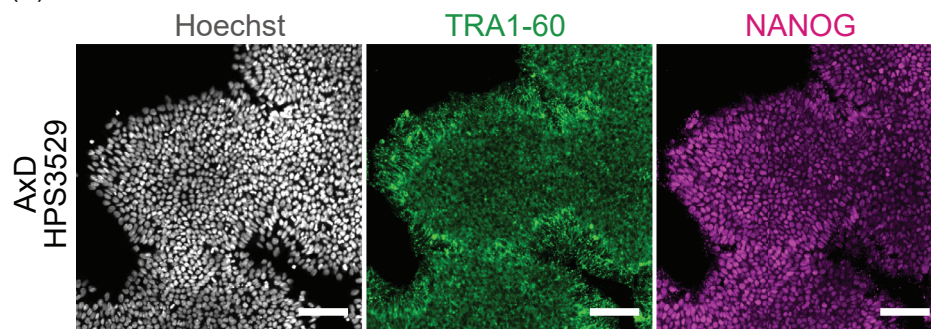

(C)

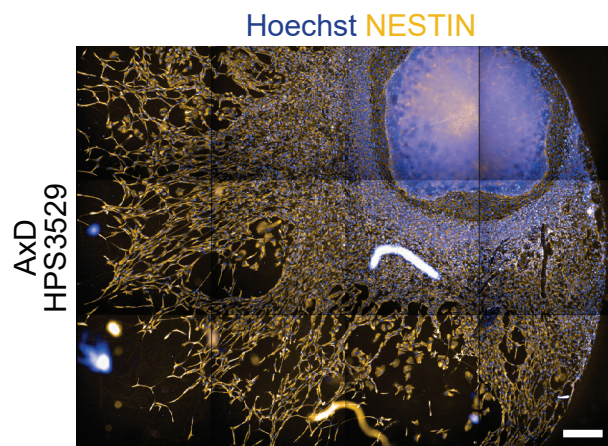

Figure S3

(A)

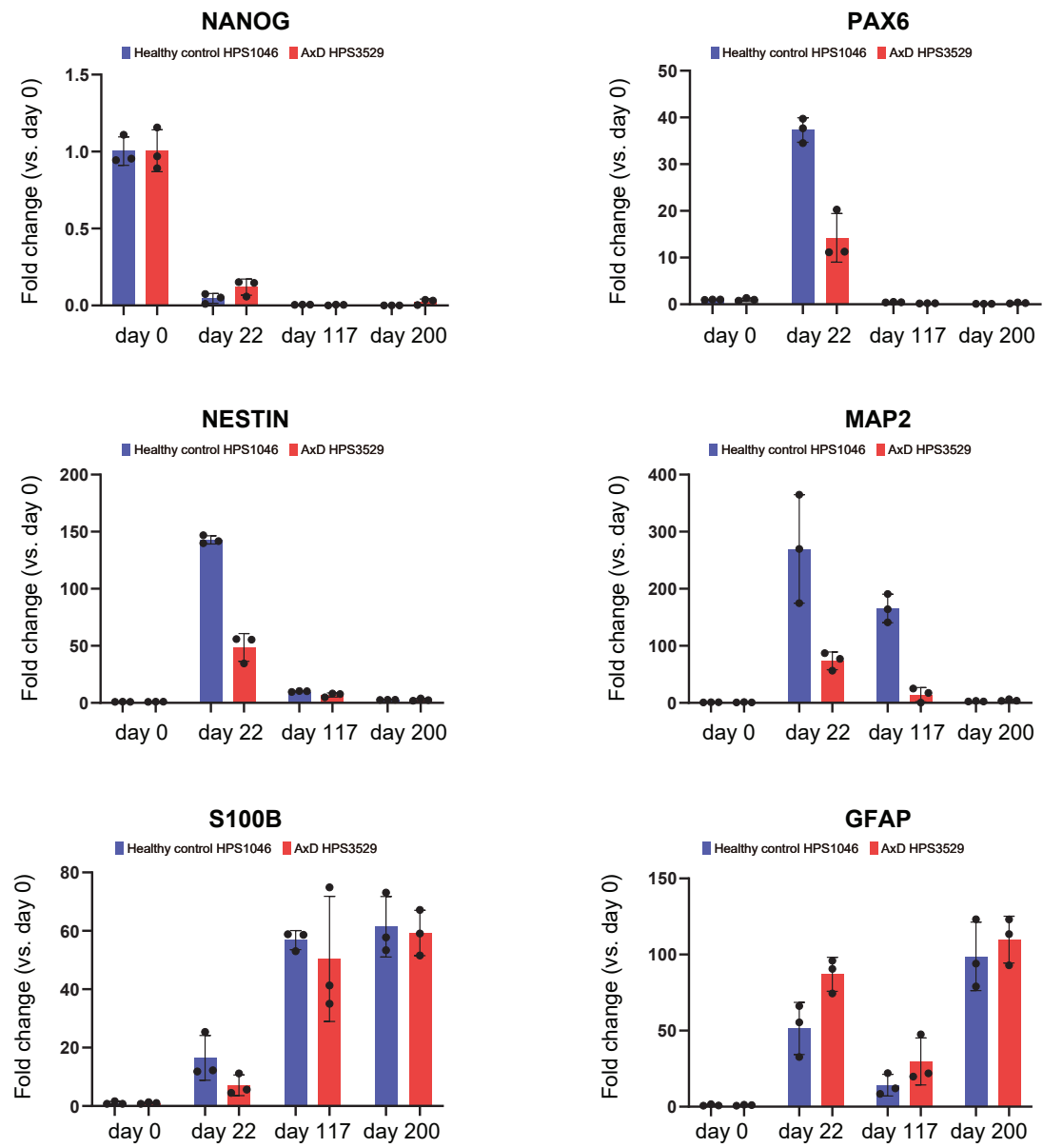

(B)

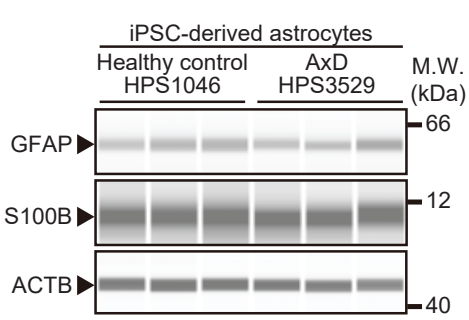

# Figure S4

(A)

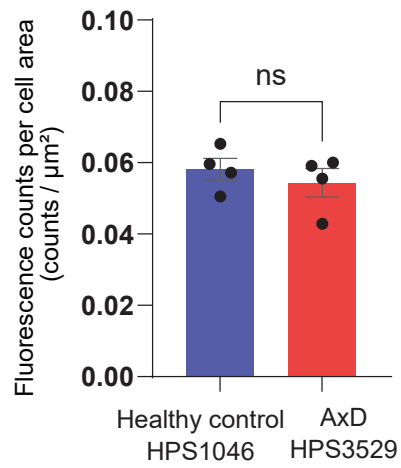

(B)

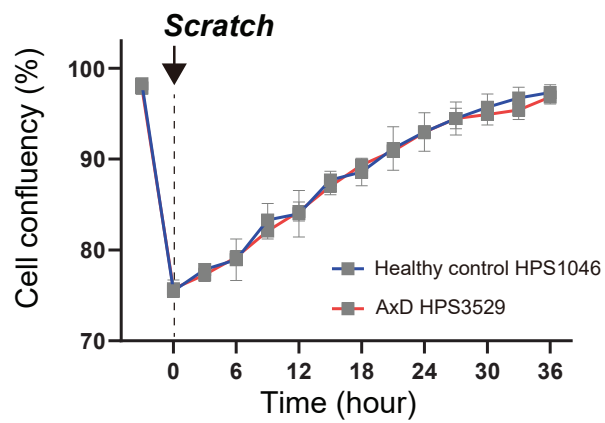

Supplement: Supplementary file 1 — Figure S1–S5 [file JCMM-28-e18214-s001.pdf]
