# Supplementary material for: Induced pluripotent stem cell‐based assays recapture multiple properties of human astrocytes
Source: J Cell Mol Med. 2024 Mar 20;28(7):e18214. doi: 10.1111/jcmm.18214 (PMC10955154; doi:10.1111/jcmm.18214)
Supplement: Supplementary file 2 — Table S1 [file JCMM-28-e18214-s002.docx]

**Table S1.** **Summary of iPSCs in this study**.

|  | **Control** | **Alexander disease** |
| --- | --- | --- |
| Cell identifier | HPS1046 | HPS3529 |
| Ethnicity | Japanese | Japanese |
| Sex | Male | Female |
| Age | 40s | 40s |
| GFAP genotype | wild | c.791_792TG>CT |
| Origin | peripheral blood mononuclear cell | |
| Reprogramming vector | episomal vector (Oct3/4, Sox2, Klf4, L-Myc, Lin28, mp53DD, EBNA1) | |
